# Oncolytic Newcastle Disease Virus as Cutting Edge between Tumor and Host

**DOI:** 10.3390/biology2030936

**Published:** 2013-07-02

**Authors:** Philippe Fournier, Volker Schirrmacher

**Affiliations:** 1 German Cancer Research Center (DKFZ), Im Neuenheimer Feld 280, 69120 Heidelberg, Germany; E-Mail: P.fournier1@yahoo.de; 2 IOZK Köln, Hohenstaufenring 30–32, 50674 Cologne, Germany

**Keywords:** RNA virus, tumor immunology, immunotherapy of solid tumors, tumor vaccination, virus, dendritic cells, danger signals, CD8 T-lymphocytes

## Abstract

Oncolytic viruses (OVs) replicate selectively in tumor cells and exert anti-tumor cytotoxic activity. Among them, Newcastle Disease Virus (NDV), a bird RNA virus of the paramyxovirus family, appears outstanding. Its anti-tumor effect is based on: (i) oncolytic activity and (ii) immunostimulation. Together these activities facilitate the induction of post-oncolytic adaptive immunity. We will present milestones during the last 60 years of clinical evaluation of this virus. Two main strategies of clinical application were followed using the virus (i) as a virotherapeutic agent, which is applied systemically or (ii) as an immunostimulatory agent combined with tumor cells for vaccination of cancer patients. More recently, a third strategy evolved. It combines the strategies (i) and (ii) and includes also dendritic cells (DCs). The first step involves systemic application of NDV to condition the patient. The second step involves intradermal application of a special DC vaccine pulsed with viral oncolysate. This strategy, called NDV/DC, combines anti-cancer activity (oncolytic virotherapy) and immune-stimulatory properties (oncolytic immunotherapy) with the high potential of DCs (DC therapy) to prime naive T cells. The aim of such treatment is to first prepare the cancer-bearing host for immunocompetence and then to instruct the patient’s immune system with information about tumor-associated antigens (TAAs) of its own tumor together with danger signals derived from virus infection. This multimodal concept should optimize the generation of strong polyclonal T cell reactivity targeted against the patient’s TAAs and lead to the establishment of a long-lasting memory T cell repertoire.

## 1. Introduction

The resistance of cancers to conventional therapies necessitates the search for new treatment strategies. The idea of using replication-competent viruses to destroy cancers is attractive to this end but is not new. Already in the mid-1950s, Newcastle Disease Virus (NDV) was reported as having oncolytic activity [1]. The clinical evaluation of this virus over several decades as anticancer reagent in various clinical settings corroborates its safety and effectivity. This is based on its tumor selective replication and oncolytic activities, allowing the virus to selectively attack tumor cells while leaving healthy cells undamaged. Another aspect that has received increasing interest is the property of NDV to activate host anti-tumor immunity. This is crucial since spontaneous antitumor immune responses are often not efficient enough due to the tumors’ inability to support an immune response or even to inhibit it through various active and passive mechanisms (tumor evasion and tumor-induced immunosuppression).

The concept of using NDV for immunotherapy has been pioneered by William Cassel with NDV oncolysate. The approach was further followed by the senior author of this review who developed the live cell vaccine ATV-NDV derived form the patient’s tumor cells by infecting them with a non-lytic strain of NDV. The discovery of dendritic cells (DCs), in 1973, revolutionalized the field of vaccination only recently with the approval in 2010 by the FDA of the first DC-based cancer vaccine [2]. We will describe how DC-based vaccination can be combined with systemic application of oncolytic NDV and also with NDV oncolysates. In this review, we will highlight the evolution over the last 60 years of therapies based on NDV and present the latest development in this field.

## 2. NDV for Cancer Therapy

**Figure 1:**
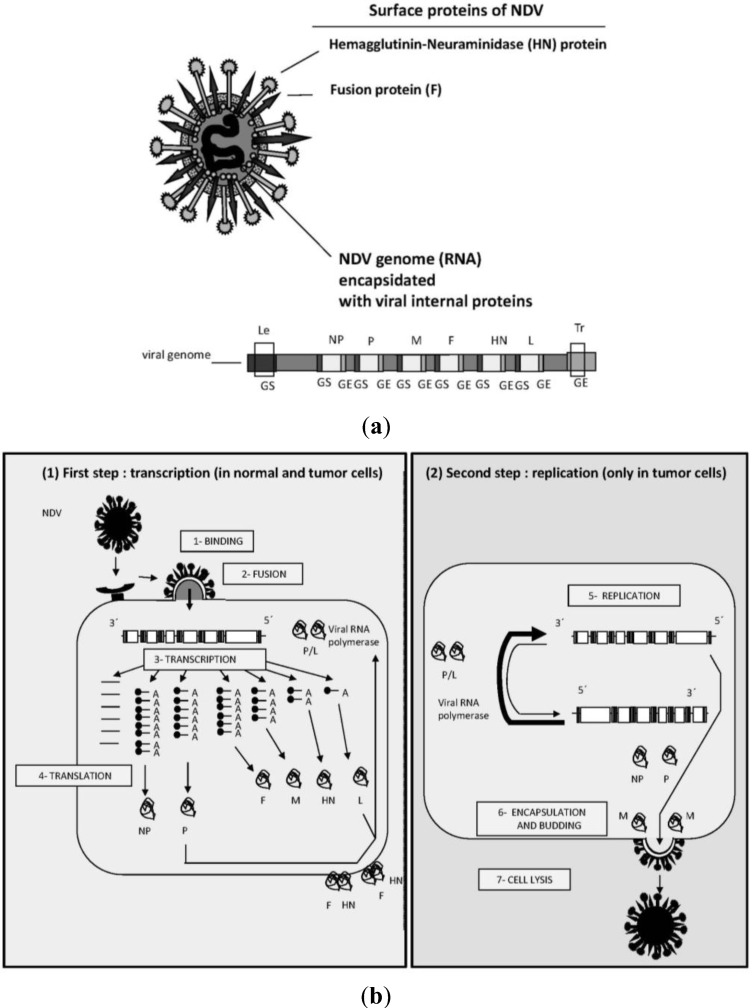
Newcastle Disease Virus: (**a**) structure;(**b**) replication *in vitro* (see the main text for more details).

NDV got its name in 1926 from an outbreak among chickens at a farm near Newcastle-upon-Tyne in England [3,4]. This virus is now classified as an avian paramyxovirus-1 (APMV-1) in the avulavirus genus of the family paramyxoviridae within the order mononegaviralis [5]. It is an enveloped virus of 100–300 nm diameter with a negative-sense single stranded RNA genome of roughly 16,000 nucleotides (Figure 1a). The RNA contains six genes encoding several major polypeptides. Among them are two surface proteins: the Hemagglutinin-Neuraminidase protein (HN, 74 kDa) and the Fusion protein (F, 67 kDa). The HN protein mediates the binding of the virus to host target cells whereas the F protein allows the fusion of the viral envelop with the cellular membrane of the target cell. 

Infection of cells by NDV can be schematically divided into two sequential steps (Figure 1b).

(i) Binding, fusion, transduction of the viral genome and transcription of viral genes: This first step involves the binding of the virus—via a lectin-like cell binding domain of the HN molecule—to ubiquitously expressed host cell surface receptors expressing distinct carbohydrate side chains (*i.e.*, α2-3 and α2-6-*N*-linked sialic acids [6]). This is followed by the activation of the fusion protein F, which is synthesized as an inactive precursor (F0, 67 kDa). During fusion, F undergoes proteolytic cleavage to yield the biologically active protein consisting of the disulfide-linked chains F1 (55 kDa) and F2 (12.5 kDa). The concerted action of HN and F leads to fusion of the viral membrane with the host cell membrane. This involves two receptor binding sites of the globular head of HN and activation of the HN stalk and of the F protein [7]. This membrane fusion event allows the viral genome to enter the cytoplasm of the host cell. There, the negative strand RNA-genome is transcribed into messenger RNAs and translated into viral proteins. The three proteins NP, P and L, which are produced in infected cells, are then used to assemble the nucleocapsid as antigenome.
(ii) Viral replication (second step): The anti-genome is then used as a template for amplification of the viral genome. Very interestingly, NDV can trigger, shortly after infection, autophagy to enhance virus replication [8]. The M protein and the envelope proteins HN and F, after post-translational modification, move to the membrane where virus assembly and budding occurs [9]. In this process, single copies of the NDV genome become wrapped into an outer coat envelope that is made from the host cells’ plasma membrane.

Among all the OVs, which have been investigated as anticancer reagents, NDV has emerged as a particularly promising agent for virus therapy of cancer [10]. This interest can be explained by three features presented by this virus: (i) tumor selective replication and safety profile (see Section 2.1), (ii) oncolytic potential (see Section 2.2) and (iii) immunostimulatory properties (see Section 2.3).

### 2.1. Tumor Selective Replication and Safety Profile

Integral to the live cycle of all RNA viruses is the formation of double-stranded RNA (dsRNA), which activates a spectrum of cellular defence mechanisms involving interferon (IFN)-α and -β. Tumors provide a relatively permissive substrate for the propagation of RNA viruses such as NDV because mutations in tumor cells often cripple the IFN system to allow un-inhibited proliferation and to provide resistance to apoptosis [11]. The first step of infection by NDV takes place in all cell types whereas the second step (which corresponds to viral replication) occurs only in tumor cells since it is stopped very rapidly in normal cells.

Through all the studies performed, NDV showed selective replication in nearly all the tested human/murine tumor cells and transformed cells [12,13]. NDV, like other viruses, has developed immune escape mechanisms in its natural host, the bird. NDV uses for this purpose the viral V protein. Once produced in the infected cells, the V protein interferes with Signal Transducer and Activator of Transcription (STAT)-mediated type I IFN signals. This immune evasion mechanism of NDV is species-restricted and does not apply to mammalian cells. Therefore, in rodent and mammalian normal (non-malignant) cells, the NDV induced type 1 IFN response is capable of preventing viral replication [12,14].

The feature of selective replication in tumor cells appears to be directly related to the lower capacity of tumor cells, in comparison to normal cells, to mount a strong type 1 IFN response. In normal cells, NDV establishes first an early anti-viral state through a functional IFN signalling cascade of transcription factors that leads to prevention of viral genome amplification and consequently to inhibition of viral replication. Tumor cells have a weaker type 1 IFN response and a weaker sensitivity to type 1 IFN-receptor (IFNR) signaling. The impairment of interferon pathways appears to be a rather common feature during tumorigenesis. That makes tumor cells highly susceptible to NDV infection and to the ensuing oncolytic events. 

An inverse correlation exists between the expression of four anti-viral genes and the susceptibility of cells [normal cells and tumor cells) to infection by NDV: (i) Retinoic acid-Inducible Gene (RIG)-I, (ii) Interferon Regulatory Factor (IRF)-3, (iii) IRF-7 and (iv) IFN-β [15]. In addition, IFNR plays a crucial role in the interferon-response feedback amplification loop. The second step of infection, namely replication using the full-length viral antigenome as template, has been shown to be prevented in non-tumorigenic cells [12]. This resistance of normal cells to NDV replication could be partially broken by knocking out IFNR expression [16].

It is of special significance that the activation of a rapid and strong type 1 IFN response by NDV in normal cells prevents virus replication, cytotoxic effects and pathology in normal tissues. This explains the high safety profile of this biologic agent. The anti-viral response of normal cells is initiated through the recognition of viral products such as dsRNA by the two types of pathogen recognition receptors (PRR): (i) Toll-like receptors (TLRs), especially TLR3 and (ii) RIG-like receptors (RLRs). Among the RLRs, RIG-I was demonstrated to be a cytoplasmic viral RNA receptor ([17] and reviewed in [18]). 

The strong type 1 IFN response to NDV involves an early and a late phase. In the early phase, RIG-I, in addition to TLRs, participates in the recognition of paramyxoviruses and orthomyxoviruses [19] whereas MDA-5, another RLR, is central for recognition of picornaviruses [20]. Very interestingly, RIG-I binds specifically to RNA containing 5'-phosphate such as viral RNA, while mammalian mRNA is either capped or contains base modifications [21]. RIG-I appears thus to be able to discriminate between self and non-self (viral) RNA [22]. Once activated, RIG-I binds to the CARD containing adaptor protein IPS-1 which, after a further signal cascade, activates, in the early phase, IRF-3. This transcription factor is then phosphorylated, translocates to the nucleus and induces IFN responses [23]. In the late phase of the IFN response, the type 1 IFN molecules secreted in the early phase interact with cell surface expressed type 1 IFN-receptors and initiate an amplification loop of the IFN response, which involves STAT proteins and IRF-7 (for a review, see [24]). 

Although NDV can induce fatal respiratory diseases in birds (e.g., chicken pest), this avian paramyxovirus, is not a human pathogen. NDV, when applied to humans, usually induces only mild fever for a day or conjunctivitis. No severe adverse effects have been reported despite applications in several thousands of people during 2 decades in Europe and the USA [11]. 

No mechanism of NDV to evade immune responses in mammalian normal cells has been observed and described. At present, there exists an extensive safety database for NDV [25]. This virus shows a high tolerability in humans. For example, when exposed to oncolytic forms, the maximal tolerated dose is around at least 3.3 × 10^9^ infectious particles when the virus is applied by the intravenous route and 4.3 × 10^12^ infectious particles by the intra-tumoral route [25]. In conclusion, it appears that NDV has advantages as a vector because of its safety and tolerability in cancer patients.

These observations can be combined with other advantageous characteristics related to the biology of the virus. In particular, the modular nature of gene transcription, the undetectable rate of recombination, and the lack of a DNA phase in the replication cycle make NDV a suitable candidate for the rational design of a safe attenuated vaccine and gene therapy vector [26]. There may be additional *in vivo* mechanisms, such as cell fusion and syncytium formation, which allow virus escape from neutralizing antibodies. The general human population is seronegative when tested for antibodies against NDV antigens [3,25]. The viral vector is not able to lead to cellular transformation. Finally, a robust virus production and a manufacturing system based on eggs is available from the traditional vaccine field. All these features make NDV a safe viral vector system for human application.

### 2.2. Oncolytic Potential

Another interesting property of NDV, which is expected to play an important role during the systemic application of the virus, is its potential to induce tumor lysis after infection. Naturally occurring NDV strains have been reported to be effective oncolytic agents in a variety of animal tumor models [27]. NDV can show strong oncolytic capacity *in vitro* and in animals when applied intra-tumorally or peri-tumorally [28,29]. The oncolytic effects lead to cell destruction as it can be observed *in vitro*. *In vivo*, such oncolytic effects seem to be limited because of the interferon (IFN) response, which blocks NDV replication quickly after virus administration. 

Like many viruses, NDV induces apoptosis by activating the mitochondrial pathway. And this is responsible for the cytopathic effect caused in cells, which are infected by NDV [30]. NDV has been demonstrated by Elankumaran *et al.* to mediate its oncolytic effects by both intrinsic and extrinsic caspase-dependent pathways of cell death [31]. In another study, NDV-induced apoptosis was shown to be dependent on upregulation of TNF-related apoptosis-inducing ligand (TRAIL) and caspase activation [32]. This causes opening of mitochondrial permeability transition pores and loss of mitochondrial membrane potential, leading to a complex mechanism forming at the end the apoptosome, which is responsible for the activation of the apoptosis process [33]. This self-sacrifice of cells is a primary mechanism of virus protection to prevent spread of the virus to neighboring cells. This defense may prove effective if cell death occurs before assembly of NDV progeny [27]. It limits the spread of infection and hinders the oncolytic effects of NDV, especially if the virus is present at low concentrations. NDV matrix (M) protein binds to Bax which contributes to a faster cell death. Other pro-apoptotic proteins upstream of mitochondria are involved as well [34]. NDV could exert oncolytic activity also against hypoxic cancer cells, thereby corroborating its potency as therapeutic agent [35].

NDV has a wide host range with at least 27 of 50 orders of birds susceptible to infection. It is categorized into three pathotypes depending on the severity of the disease that it causes in birds: (i) lentogenic (avirulent), (ii) mesogenic (intermediate) and (iii) velogenic (virulent) [3]. Lentogenic NDV does not cause overt pathology in adult birds and is considered of low virulence. Viruses of intermediate virulence cause respiratory disease. Among the highly virulent velogenic NDV isolates, there are viscerotropic forms marked by lesions of the digestive tract, whereas neurotropic forms are characterized by respiratory and neurological problems. Lentogenic strains behave as non-lytic whereas velogenic strains are lytic (cytotoxic). The more virulent NDV strains have a furin cleavage site in their F protein. This site allows F activation in a proteolytic environment such as the tumor microenvironment. This leads to multicyclic viral replication, syncitium formation and cross-infection from one tumor cell to another. The property of the released progeny virus either to be infectious or non-infectious depends on the virulence of the NDV strain. Cytopathic effects of lytic NDV strains can be seen by formation of plaques in tumor cell monolayers (plaque assay, see [36] for an example) or in tissue sections (tissue plaque assay). Hydrophobic fusion peptides within the viral fusion protein promote syncytium formation between infected tumor cells whereby the virus spreads without an extracellular phase, leaving an oncolytic plaque. The killing potential of lytic NDV strains is remarkable. Such strains have been shown to have a high capacity for killing tumor cells. One infectious particle leads *in vitro* to the death of approximately 10,000 cancer cells in 2–3 days.

One major difference between lytic and non-lytic strains is that lytic strains are able to produce infectious progeny virus particles in human neoplastic cells, whereas non-lytic strains are not [37]. Consequently, non-lytic strains can only perform a monocyclic replication cycle in tumor cells. The reason is that the progeny virus particles made by non-lytic strains contain only an inactive precursor from the F protein. Advantages of the lytic properties of some NDV strains is that the production of infectious progeny virus particles, after a first round of viral infection, gives them the ability to spread the virus in tumor tissues via multicyclic replication. For non-lytic and lytic NDV, it has to be noted that the virus replicates quickly and efficiently in human cancer cells, leading to the exponential increase of the viral particle number. The oncolytic potential of NDV can explain the direct anti-tumor activity of this biological agent.

### 2.3. Immunostimulatory Properties

NDV has been shown over the last 2 decades to possess interesting immunostimulatory properties. One associated important feature is its capacity to induce large amounts of type 1 IFN when in contact with human peripheral blood cells. This is linked to the nature of the dsRNA structures, which are produced within the cytoplasm during the viral replication, thereby provoking a strong cellular IFN response. 

The viral surface HN protein has also been shown to be an important element for the immunostimulatory properties of this virus. NDV infection of tumor cells modifies the tumor cell surface (increased expression of the viral HN and F proteins after about 10 hours [38]). Through their receptor binding activity, the HN proteins introduce new cell adhesive strength for lymphocyte interactions [38,39] and T cell co-stimulation [40]. In addition, human tumor cell infection by NDV leads to up-regulation of HLA and ICAM-1 molecules. Further events are induction of IFNs, chemokines (IP10, RANTES) and finally apoptosis [41]. Double-stranded RNA (dsRNA), a by-product of viral replication, can activate cytoplasmic PKR [42] and RIG-I [43] as well as endosomal TLR3 [44]. 

All the danger signals, which derive from the infection by NDV (dsRNA, IFN-α, HN cell surface protein) [45,46], allow activation of multiple innate immune responses (by monocytes [47,48], DCs [49] and NK cells [50]). They also play an important role during the presentation of tumor associated antigens (TAAs) to T cells. Such co-signals are important for the induction of an effective adaptive immune responses against the tumor including CD4+ and CD8+ T cells [40,51] (summarized in [39,52]).

NDV, as a strong inducer of type 1 IFN, is expected to have a strong effect on the immune response, given the recently appreciated role of type 1 IFNs in anti-glioma immunosurveillance in mouse studies [53]. The pro-inflammatory context, which is induced upon the addition of the virus NDV, may be able to break tumor tolerance, as it has been shown with a T cell clone *in vitro* [54].

In contrast, other viruses, which have adapted to the mammalian immune system, have developed virally encoded inhibitors of immunity such as TAP-inhibitors, cytokine decoys, micro-RNAs and viral proteins that antagonize type 1 IFN induction [55].

**Figure 2:**
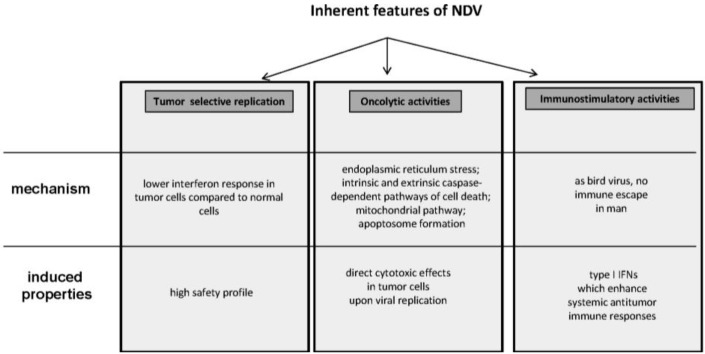
Newcastle Disease Virus: its three main inherent features (see the main text for more details).

In conclusion, NDV presents three key features—tumor selective replication, oncolytic capacity and immunostimulatory properties—which make it a promising agent to be developed for cancer therapy in man (Figure 2).

## 3. Experience with NDV in Preclinical and Clinical Studies

The concept of using viruses in tumor therapy dates back to the early 1900s when observations were made of remissions of human cancers following natural viral infections [56,57] (e.g., with mumps, measles virus or influenza). For example, in the case of leukemia, it was recognized that contraction of influenza produced beneficial effects. However, no cases were reported where an accompanying infectious disease led to complete cure of cancer. Such observations, as well as the lack of successful anti-cancer reagents, have repeatedly stimulated interest in using viruses for cancer treatment during the past hundred years. Intentional application of viruses to humans for the treatment of cancer began already in the 1950s.

Many of the different viruses, which were tested for clinical application, caused problems because of side-effects, in particular neurotoxicity. In addition, most viruses became very quickly ineffective after the first *in vivo* injection because of generation of neutralizing antibodies. Among all those viruses, NDV emerged as one of the first oncolytic agents with promising properties. This bird virus, since then, occupied a central place among other viral candidates as anti-cancer reagent. 

However, the interest in this virus has fluctuated over the last decades, reaching a first peak in the 1950s and 1960s (see Section 3.1), followed by a near-neglect in the 1980s and a resurgence of interest in the past two decades (see Section 3.2). 

### 3.1. Pioneering Studies (1950s–1970s)

Early pioneering preclinical studies based on the oncolytic properties of the NDV strains *Sato* and *Miyadera* were performed in the 1950s using Yoshida sarcoma in rats with encouraging results [58]. Preliminary clinical trials with NDV were rather anecdotic. The first application of this virus to humans dates back to the study by Wheelock and Dingle [59] in 1964. They published their observations on a patient, who was treated by repeated injections of NDV in an attempt to affect his acute leukemia. Another study by Cassel and Garrett [60] was performed in a previously untreated woman with inoperable cervix carcinoma. A single intra-tumoral NDV injection resulted in extensive sloughting of tumor tissue and regression of lymph node (LN) metastases, with considerable subjective improvement. However, the patient expired 7 months later. William Cassel, who described already in 1965 the anti-neoplastic properties of NDV [60], developed the oncolytic substrain *NDV 73-T* to treat cancer patients. Derived from a field isolate, this strain is neurovirulent in young birds. It kills 1-day-old chicks by paralysis after intracerebral inoculation. But in older birds, it leads, after intramuscular administration, to viral replication without any manifestion of pathology or disease. This suggested its safety for human application. The potential oncolytic activities of this NDV strain was shown by its ability to lyse ascites tumor *in vivo* in Ehrlich tumor-bearing mice. Intraperitoneal injection of a critical intermediate dose of this NDV cured tumors, whose cells had been applied 6 days before into the mice. No ascites developed during a 2-month observation period thereafter. The life of mice with tumors older than 6 days when receiving *73-T* treatment was prolonged compared with untreated tumor-bearing controls. In addition, this strain was observed to spare normal tissues [1,60,61].

In Hungary, Laszlo K Csatary worked with a veterinary vaccine strain of NDV [62,63,64,65]. This virus strain corresponds to an attenuated lytic variant that was generated by several passages in chicken embryos of the original Hertfortshire strain of NDV designated *Herz’33* [66]. Studies have demonstrated a remarkable genetic stability, even after prolonged passage. 

Csatary’s concept was to use this strain as inhalation vaccine for systemic treatment and non-specific immune stimulation based on the strong capacities of the virus to activate host immune responses in terms of cytokine production. It was entered for clinical evaluation in Hungary, where an attenuated variant, designated as *MTH-68/H* (produced under current Good Manufacturing Pratices (cGMP) conditions) was administrated weekly for 6 months by the inhalation route to patients with advanced cancers [67]. Laszlo K. Csatary, at the time in Hungary, reported in 1993 the results of a prospective phase II trial in Hungary, in which 33 patients with advanced cancers were treated [64]. Fifty-five percent (18 out of 33) of the patients, primarily those with colorectal cancer, responded to treatment compared to 8% (2 out of 26) of patients who did not receive virus treatment. After 2 years, there were seven survivors in the treated group compared to none in the control group. No systematic clinical studies were followed up.

In the 1980s, the development of chemotherapeutic reagents brought new hope for cancer treatment. And virus treatment was not “en vogue” anymore during these years. But the continuous problems with the development of tumor resistance to treatment with chemotherapeutic agents, which resulted in cancer relapses, eventually led to a regain of interest from clinicians for OVs as tumor cell killers during the past two decades (as highlighted by an editorial in 1999 [68]).

### 3.2. Regain of Interest in NDV (1990s–2010s)

The interesting clinical observations, which had been made in the past with the NDV strains *73T* and *MTH-68*, stimulated further evaluations of their anti-cancer properties. To this end, genetically immunosuppressed SCID mice carrying xenografted human neuroblastoma or fibrosarcoma were used. These mice responded well to a single intra-tumoral injection of the NDV *73T* virus strain and showed complete or partial remission [69,70]. Based on their very promising data obtained with this NDV strain in mouse models with xenografted tumors [69,70], Lorence and collaborators performed clinical trials using a mesogenic NDV isolate called *PV701,* which was derived via plaque purification from the naturally attenuated vaccine strain *MK107* [71]. 

A first Phase I clinical trial investigated the safety aspects of *PV701*. It showed for 79 patients with various advanced solid cancers [72] that intravenous application of single or multiple doses from 1.2 × 10^10^ to 1.2 × 10^11^ pfu were well tolerated with only grade 1 and 2 toxicity (fatigue, flu-like and gastrointestinal syndroms as most frequent side-effects). Several dose regimens were evaluated. It was also observed that initially giving lower virus doses permitted escalation of doses later (desensitization procedure). This allowed a reduction in some cell toxicity, especially at the tumor site. Of the 79 patients, one partial remission was observed and 14 patients showed no tumor progression between 4 and 30 months after therapy. The most common adverse events were fever and other flu-like symptoms.

In total, the obtained results after systemic application of NDV were quite disappointing [72,73,74,75] because of the difference between the spectacular activity of NDV in animal systems, where complete eradication of certain selected xenotransplanted human tumors was observed [69,70] and its poor performance in human clinical trials. The xenotransplant animal systems appeared then not as reliable predictors of clinical responses, although they indicated that intra-tumoral injections of NDV showed much more therapeutic effects than systemic virus administration [76]. 

A Hungarian group studied the use of the NDV strain *MTH-68/H* as a treatment modality for patients with various cancers. They reported on a case series of 4 patients and on a phase II trial. There was some benefit to some patients with glioblastoma after repeated *i.v.* applications of this oncolytic NDV strain [65,77]. For example, treatment of a 14-year-old boy with glioblastoma induced a partial response, which was documented by MRI but without histological proof [77]. But the anti-tumor response, which was claimed as responsible for the tumor regression, was supported only by anecdotal case reports. Some other clinical trials based on systemic administration of the lentogenic NDV strain *HUJ* were performed at the Hadassah Medical Center (Jerusalem, Israel). From the involved patients suffering from glioblastoma multiforme (GBM) and receiving systemic application of the *HUJ* virus strain, one patient has been reported to have achieved a complete response [78]. 

From all these clinical studies, it appears that an effect of NDV as anti-cancer agent can be observed, even when applied to patients suffering from advanced cancers. But the data obtained with oncolytic NDV were not as good as one had hoped for. 

## 4. NDV as Adjuvant of Tumor Vaccine

This concept is based on using NDV as biologic adjuvant for an effective tumor vaccine knowing that virus-induced augmentation of the antigenicity of tumor antigens has been observed in several model systems [79,80]. It is based on the combination in a vaccine of a specific component expressing the tumor antigens (tumor cells) with a non-specific component (NDV) which increases its tumor immunogenicity in order to induce a strong anti-tumor immune response. 

**Figure 3:**
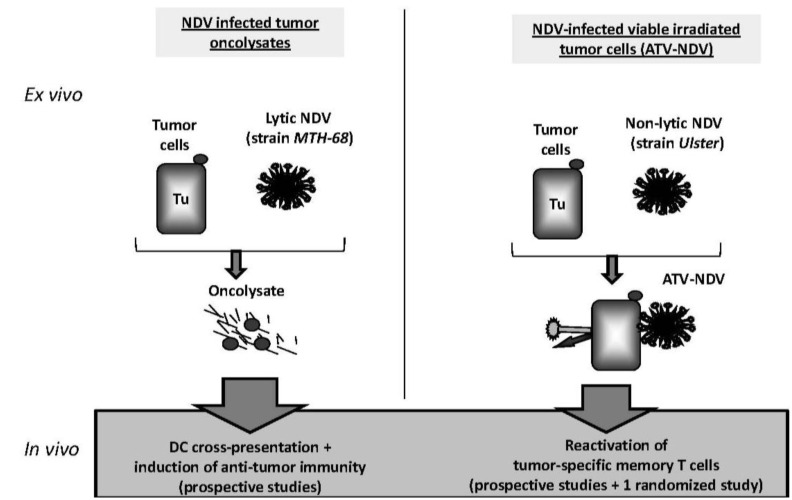
Vaccination with Newcastle disease virus (NDV)-infected tumor cells: the two main strategies(see the main text for more details).

Two main strategies for active specific immunotherapy (ASI) including NDV—respectively as lytic or non-lytic virus—as adjuvant were evaluated in clinical trials (Figure 3).

### 4.1. Immunotherapies with NDV Oncolysate

To stimulate the anti-tumor immune responses, William Cassel *et al.* developed already in 1977 a second direction based on tumor oncolysates. These were obtained from melanoma upon infection with the oncolytic NDV strain *73-T* [81] to induce anti-tumor immune responses [82]). The rationale was that viral oncolysates combine the anti-tumor effects of post-oncolytic immunity with active tumor-specific immunization. To have a broad repertoire of melanoma-associated tumor antigens, William Cassel selected three melanoma cell lines. One allogeneic cell line was substituted with autologous melanoma cells when available. Oncolysates were produced by infection with NDV without any additional chemical or biological adjuvant. The vaccinations were performed routinely for all included patients as long as they lived. 

The trial gave an unprecedented over-60% survival rate after 10-years follow-up compared to only 33% of comparable non-vaccinated patients [83]. From all the clinical studies conducted by William Cassel *et al.* [82,83,84], there was a strong claim that oncolysates prepared with NDV eliminate subclinical microscopic disease left behind after surgical removal of tumors. Since these trials were evaluated against historical controls and were not prospectively randomized, the real effects of the vaccine cannot be assessed. Nevertheless, no growth enhancement in patients, which were treated with viral oncolysates, was observed. And relapses, if they occured, tended to be delayed in appearance and protracted in course ([83], reviewed in [84]).

### 4.2. ATV-NDV: Live Autologous Virus Infected Tumor Cell Vaccine

The concept of exploiting the immunostimulatory properties of NDV to augment the immunogenicity of tumor cells was further developed by the senior co-author of this review An autologous live cell tumor vaccine, called ATV-NDV, was obtained upon modification of patient’s tumor cells via infection with a lentogenic strain (*Ulster*) of NDV and γ-irradiation [39]. The term ATV-NDV attributed to the vaccine stands for autologous tumor vaccine (ATV) modified with NDV. The reason for using a non-lytic virus strain was to create a live cell vaccine rather than an oncolysate to re-stimulate anti-tumor memory T cells via direct tumor antigen presentation. The rationale was based on own findings that a certain threshold of anti-tumor immune memory can play a decisive role for the control of residual tumor cells after primary operation and in an adjuvant situation [85]. The lentogenic *Ulster* virus shows an abortive monocyclic replication cycle in tumor cells [38]. The virus progeny, which are produced, are non-infectious and the infected tumor cells, dye via apoptosis [41]. For preparation of ATV-NDV, tumor cells were isolated from freshly operated tumor specimens by mechanical dissection and enzymatic dissociation, and enriched by Percoll centrifugation. Tumor infiltrating lymphocytes (TILs) were then removed by immunomagnetic beads. The tumor cells were then frozen in aliquots that contained 10^6^–10^7^ cells. NDV infection was initiated by one hour adsorption of the virus to the tumor cells and then the cells were inactivated by 200 Gy-irradiation. This treatment inhibits the tumor cells proliferation capacity via DNA cross-linking while it does not inhibit the RNA virus from replicating in the cells’ cytoplasm. The virus modified tumor vaccine was injected intradermally. *In vivo* viral replication within the tumor cells at the site of vaccine application takes approximately 6–40 hours [38], a time sufficient to generate Delayed Type Hypersensitivity (DTH) skin responses, which are dependent on antigen-specific memory T cells. 

It is well established that tumor immunogenicity requires more than tumor antigenicity. And modification of tumor cells by infection via NDV was shown to increase its immunogenicity for T cells (CD4+ and CD8+, in particular pre-existing tumor-reactive memory T cells from cancer patients) and also allowed for activation of multiple innate immune cells (NK cells, macrophages, monocytes and DCs) [86,87]. 

In 1986, it was reported for the first time that post-operative immunotherapy with ATV-NDV prevented metastatic spread in a mouse tumor model [86]. In such experiments, post-operative vaccination with the NDV-modified irradiated tumor cell line ESb was able to cause protection from metastases in about 50% of syngeneic mice [87]. The surviving animals developed long-lasting protective immunity specific for this tumor cell line. The induced tumor immunity did not cross-protect against other syngeneic or allogeneic tumor cell lines, indicating that the anti-tumor effects are based on tumor-specific immune T cell memory. These observations were confirmed in other metastasing animal tumors such as murine B16 melanoma, 3LL Lewis lung carcinoma and guinea pig L10 hepatocarcinoma. 

In 1990, von Hoegen *et al.* described for the first time that the induction of type 1 IFN by NDV potentiates tumor-specific CTL activity [51]. The application of neutralizing anti-type 1 IFN antibodies to mice potently reduced their capacity to prime CTL precursors while, in *in vitro* MLT-CTL cultures, they reduced the generation of CTL activity.

The introduction of danger signals into tumor cells via NDV infection appeared able to break T cell tolerance to tumor antigens. For example, it could be demonstrated in an autologous human anti-melanoma CD4+ T helper clone that infection by NDV of autologous melanoma cells caused a T cell co-stimulatory activity [54]. In contrast, without NDV infection, contact with autologous melanoma cells rendered the CD4 T cell clone non-reactive and un-responsive, even to subsequent stimulation by IL-2. Further results relating to mechanism of function of NDV in the immune system have been reviewed in [88].

The first description of clinical results following post-operative vaccination with ATV-NDV modified tumor cells was reported for primary operated breast cancer patients in 1996 [89]. In this phase II trial, the 5-year survival rate in vaccinated patients was more than 30 per cent higher than in a comparable group. Tumor cell number and viability were defined in this study as quality and efficacy parameters of the vaccine. Some other results were reported in 1998 in a review summarizing all the hitherto performed studies about immunization with virus-modified tumor cells [39]. Two-year survival rates were improved in comparison to standard treatment in colorectal carcinoma by 24% when the disease at diagnosis was locally advanced [90] and by 26% with R0 resected liver metastases [91]. Similarly, 2-year survival rates in recurrent melanoma were improved by 20% [40]. Even in a disease as bad as glioblastoma multiforme, the median overall survival (OS) of 23 vaccinated patients was twice as long as that of a non-vaccinated control group involving 87 patients from the same hospital. There was one complete remission of remaining brain tumor after operation and several long-term surviving patients, who had developed a specific anti-tumoral long term memory [92].

Data from 5 years’ observations are also available for colorectal carcinoma patients. OS in vaccinated colorectal carcinoma (locally advanced) was increased by ATV-NDV: 61% of the patients were still alive after 5 years, which compares favorably with the expected 39% under standard therapy [89]. OS at 5 years in vaccinated breast carcinoma (locally advanced) was improved by 36% [89]. OS in vaccinated Head and Neck Squamous cell carcinoma (HNSCC) was improved by 23% [93]. 

Results from a randomized-controlled Phase II/III study were as follows. Patients with histologically confirmed liver metastases from colon cancer or rectum cancer were randomized to an ATV-NDV vaccination or to a control arm [94]. After complete resection of the liver metastases, patients randomized to the vaccination arm received six vaccinations during the first 6 months. After a follow-up period of more than 10 years, there was still a significant advantage for vaccinated colon cancer patients with respect to OS (*p* = 0.042) and with respect to metastases-free survival (*p* = 0.047). In contrast, in rectal carcinoma patients, the same treatment with ATV-NDV did not influence OS, recurrence free survival (RFS) or metastases-free survival. This difference in responsiveness to ASI between patients with two different tumor entities is of biological relevance but not yet understood. That this difference came out so clearly and that the follow-up period was much longer than in other published studies underlines the high quality of the trial. For colon cancer patients, there is thus a good future prospect with the ATV-NDV vaccine. The trial indicates the value and potential of cancer vaccines using NDV-infected cells. 

A further study with higher numbers of patients is warranted and further investigations into the implied mechanism, efficacy and long-term immunity are required.

In conclusion, it can be stated that clinical efficacy in terms of survival has been observed when NDV was used as adjuvant in strategies of active specific immunotherapy—that is when NDV and tumor cells were combined together before application to cancer patients. This vaccine has been evaluated from 1989 to 2008 in at least ten Phase II trials and one randomized, controlled Phase II/III study. Positive effects on long-term survival of cancer patients upon post-operative anti-tumor vaccination with ATV-NDV have been summarized and discussed [85]). The results suggest either the direct involvement of the tumor cells of the vaccine in antigen presentation or the importance of prolonged expression and release of TAAs with danger signals derived from viral infection of the tumor cells. Before the discovery of DCs and the general acceptance of the concept of antigen cross-presentation, the above conclusion was regarded as the obvious explanation. Today an additional role of DCs in taking up the vaccine cells or viral oncolysate for cross-presentation of TAAs to T cells needs to be considered.

## 5. DC Based Vaccines

DCs expressing tumor material of the patient’s own cancer are known to be capable of inducing a Th1 tumor-specific immune response. But it took a long time from DC discovery to their clinical application. One reason is that the number of circulating DCs in the blood is extremely low. That hindered clinical application of a DC therapy for a long time. Within the last 2 decades, new insights into the biology of DCs together with new developments in biotechnology have opened the possibility for generation of DCs outside the body. Starting from circulating white blood cells and employing speciﬁc culture conditions, it is now possible to generate *ex vivo* large quantities of DCs.

### 5.1. Discovery of the DCs

DCs were first described by Ralph Steinman in 1973 when observing cells from mouse spleen that adhered to glass or plastic surfaces. In addition to mononuclear phagocytes, granulocytes, and lymphocytes, a subpopulation of cells with striking dendritic shape was noticed [95]. Nearly 40 years after their discovery, DCs have been recognized as important by the award to Ralph Steinman of the Nobel Prize for Medicine or Physiology in 2011 [96]. They are particularly frequent in tissues forming an interface with the external environment, such as the skin and lung [97]. The plasticity of these cells allows them to determinate a particular DC function according to the encountered signals. The type of adaptive immune response depends mostly on the local micro-environment and on the interaction between DCs and pathogen-associated molecular patterns (PAMPs) or damage-associated molecular patterns (DAMPs). The pattern-recognition receptors (PRRs) can be the Toll-like receptors (TLRs) [98] and the RIG-I-like receptors (RLRs) ([99], among them, RIG-I). Through the innate recognition of danger signals, DCs appear, via their capacity to detect danger signals and to induce an immune response, as a critical link between innate and adaptive immune responses.

All the characteristics exhibited by DCs make these cells unique candidates for immunotherapy aiming at inducing effective T cell-mediated anti-tumor immunity. As first reported by Finkelman and colleagues in 1996 [100], DCs can present antigen *in vivo* in an immunogenic or tolerogenic fashion. They take up antigens and efficiently process them for their presentation as peptides in association with MHC molecules (either class-I or class-II) to respectively CD8 and CD4 T cells. MHC class-I molecules present self- or pathogen-derived antigens that are synthesized within the cells, whereas exogenous antigens derived via endocytic uptake are loaded onto MHC class-II molecules for presentation to CD4+ T cells. DCs are also specialized to process exogenous antigens into the MHC class-I pathway for presentation to CD8+ T cells. This process, known as cross-presentation and shown for the first time in 1976 [101], provides a mechanism that can drive DCs to generate either tolerance to self-antigens or immunity to exogenous antigen. In the steady state, non-activated DCs present self-antigens to T cells, which leads to tolerance. It was demonstrated already in 1998 by Albert and colleagues that immature DCs phagocytose apoptotic cells via integrins and CD36 and cross-present antigens to CTLs [102]. DCs are also essential for peripheral tolerance, partly through activation of Tregs as shown for the first time by Steinmann *et al.* [103]. In order to become immunogenic, DCs need to perceive danger signals, since in their absence, they will perform tolerogenic functions. Once they are activated, antigen-loaded DCs are geared towards the launching of Ag-specific immunity leading to T cell proliferation and differentiation into helper and effector cells with unique functions and cytokine profiles. 

The design of an efficient anti-tumor vaccine is influenced by an important paradigm shift in the field of immunology regarding the regulation of immunity. Indeed, a new concept has emerged that proposes that the regulation of immunity and tolerance is not only determined by the specificity of immune T cells as previously thought, but also by the context in which the antigens are presented to the immune system. This new hypothesis originally presented by CA Janeway Jr. [104] suggested that the immune system evolved to discriminate infectious non-self from non-infectious self. The implications are that, in the absence of appropriate inflammatory reactions, the self (tumor) antigens presented by DCs, will not lead to T cell activation. Therefore, a successful anti-tumor immunity will develop only in situations, in which DCs are processing tumor antigens in the presence of a pro-inflammatory environment. This could be confered by “danger signals” brought via the use of foreign organisms such as a virus or bacteria. 

The danger signal hypothesis by Polly Matzinger [105] allowed a better understanding of the induction of immune responses. Over the years, multiple protocols have been developed for *in vitro* generation of mature DCs and for their genetic modification, both through viral and nonviral approaches, to provide the three signals (signals 1, 2 and 3), which are required for T cell activation [106]. Signal 1 is crucial because it gives the specificity to the immune response. Signal 2 (co-stimulation) is necessary since, in its absence, antigen-specific T cells will become anergic. Signal 3, which is established by the local cytokine milieu, is also crucial since it influences T cell polarization. This signal is mandatory for the induction of a strong and efficient anti-tumor cellular response. It is thought to be most important for generation of cell-mediated tumor therapy and requires a Th1 (cellular) polarized response [107,108]. In particular, type 1 IFNs (IFN-α and -β) and IL-12 have been demonstrated to serve as “signal 3” for optimal CD8+ T cell expansion [109,110]. They have also been implicated in the differentiation of effector CD8+ T cells for breaking immunological tolerance. And this is essential to permit immune responses against “self” tumor antigens. 

When all the necessary signals are present, the interactions between DCs and T cells in tumor-draining lymph nodes and in bone marrow lead to T cell activation, clonal expansion, and differentiation into effector and memory cells. To initiate such immune responses, cells from the innate immune system (NK cells, NKT cells and activated macrophages) play an important role, although the exact role of these immune effector mechanisms in protecting individuals from tumors is not well defined. An alternative mechanism, referred to as “cross-dressing”, has been shown to exist in mice in a context of viral infection, by which an Antigen Presenting Cell (APC) can transfer antigen via direct contact from its surface MHC-peptide complexes to another APC without the need of further processing [111].

And this was shown to boost the memory [112] but also to prime naive [113] CD8+ T cell responses. This might be critical since the existing tumor-specific memory repertoire requires activation (reprogramming) from non-protective immune cells toward protective IFNγ-secreting Th1 cells. The immune effects of exogenous DC vaccination have been demonstrated to be contingent on the transfer of antigen to endogenous DCs but not B cells. Such antigen transfer is not due to antigen diffusion, but rather to DC-DC molecular transfer [114]. Very interestingly, the selective ablation of endogenous lymphoid-resident DCs abrogated T cell responses following DC vaccination, demonstrating the pivotal role of this subset of DCs in this phenomenon [115].

### 5.2. DC-Based Vaccine

Very quickly after the discoveries of the DCs, Steinman and colleagues demonstrated that DCs are able to mature and that they acquire novel functions following microbe encounters (reviewed in [116]). But the interests for these cells as critical players in the antigen-speciﬁc activation of naive T cells gained all its importance only 20 years ago. At that time, Kayo Inaba *et al.* demonstrated that isolated DCs pulsed *ex vivo* with antigen could, after injection into mice, elicit an immune response against cells bearing the same antigen [117]. Two years later, Steinman and Inaba described for the first time a method for generating large numbers of DCs *in vitro* [118]. This promoted experimental studies on DC biology and made their applications in clinical research easier. In 1996, Hsu *et al.* published the ﬁrst clinical trial on DC vaccines in B cell lymphoma patients. In this study, all the four patients developed measurable anti-tumor immune responses [119]. Two years later, Nestle and colleagues reported similar observations with solid tumors [120]. Based on the promising early results from these first two clinical trials, DCs have, since then, been used to treat patients with several different malignancies. Data from these clinical trials have suggested that such therapies may delay tumor progression and prolong the survival of patients with advanced cancer [121,122]. However, their activity in inducing tumor regression was limited. The rates of clinical responses rarely exceeded 10 to 15%.

Thereafter, DC-based tumor vaccines have been extensively explored, and have become attractive tools for cancer immunotherapy. They have been tested for the treatment of more than 20 malignancies, most commonly melanoma, renal cell carcinoma, prostate cancer, and colorectal carcinoma [123,124]. Meanwhile, over 150 clinical trials testing the anti-tumor activity of DC vaccines have been reported with *ex vivo* generated DCs. These were employing diverse methodologies for DC propagation, antigenic loading, and administration [125]. 

From the plethora of DC-based vaccine trials, several generalizations can be made. The first concerns safety and feasibility of this approach. The second concerns the efficacy of DC-based vaccines. Data in humans showed that such therapies were able to induce T cell responses, although not in all the vaccinated patients. However, objective clinical responses were anecdotal and concerned a minority of the vaccinated cancer patients. Although the vaccination may delay tumor progression and improve survival for some patients with advanced cancer, its capacity in inducing tumor regression was limited in general when considering the whole population of vaccinees. And at that time, DC-based vaccines largely failed the expectation of being an effective means of treating cancer. 

The limited success may be attributed to a variety of factors regarding the preparation and administration of the vaccine, the disease stage of participants in the trials, the heterogeneous nature of most tumors rendering the clinical analysis difficult, the limited number of tumor target antigens present within the DC vaccine and the context of immuno-suppression. Most of the clinical studies have been performed in immunocompromised patients with advanced disease, which are known to have dysfunctional DCs [126,127]. 

In addition, the existence of immuno-suppressive factors in the tumor micro-environment including the production of inhibitory cytokines (IL-10, TGF-β, VEGF and IL-6), the activation of STAT3, the expansion of Treg cells, and the significant suppressive effect of myeloid derived suppressor cells (MDSCs) have been shown to create a tolerogenic milieu [128,129,130]. It is now well known that the DC properties (immunogenic or tolerogenic) are affected by the micro-environment in which they are placed [123,124].

One other explanation why only sub-optimal immune activation was achieved could be linked to some observations which suggest that only a small proportion of the injected antigen-loaded DC ever reach the LNs [131,132] where priming takes place. That correlates with recent studies performed in mice which showed that the *ex vivo* antigen-loaded DC do not directly prime T cells but they shuttle their antigen to the draining LN where the antigen is acquired by resident DCs that subsequently prime T cells [133]. The dependency on endogenous cells of antigen-loaded DCs vaccines may appear as a key underlying explanation for the poor vaccine efficacy, especially considering that the cancer patients with late disease stage are likely to have an immuno-suppressive tumor environment, rendering the endogenous cells ineffective. 

Among all the clinical studies performed with DCs, two major results marked the development of the DC field as explained in the two following paragraphs. 

#### 5.2.1. Disappointment about DC-Based Vaccine (1990–2010)

In 2006, DC vaccines suffered a major setback in a phase III clinical trial, which was performed in advanced melanoma patients [134]. It was a large multicenter randomized clinical trial, which tested the administration of matured DCs compared to the standard chemotherapeutic agent dacarbazine (DTIC) in patients with metastatic melanoma. In this clinical study, DCs, which were generated in GM-CSF and IL-4 containing culture medium, were matured with TNF-α, IL-1β, IL-6 and prostaglandin (PG) E2, pulsed with a mixture of 20 MHC class-I and II-binding TAA-derived peptides and administered subcutaneously. The study failed to demonstrate that the antigen-loaded DC vaccine was superior to DTIC. The initial enthusiasm for DC vaccination thus gradually wore off. Nevertheless, many immunologists hold that DC vaccination remains a promising therapy for cancers. With a deeper understanding of DC biology, anti-tumor immunity, and tumor immune escape mechanisms novel and improved DC-based vaccines are, since then, being developed under the guidance of clinical feedback. 

#### 5.2.2. The Second Birth of DC Therapy (2010-today)

The breakthrough came in 2010. The approval by the US FDA of the ﬁrst DC vaccine, named Provenge (or Sipuleucel-T), for the treatment of prostate cancer [2], was a milestone in the immunotherapy ﬁeld of DC vaccines. Realizing the long-pursued dream of cancer vaccines to mobilize the patients’ own immune system against tumors, this treatment appeared as a prototype for development of anti-tumor vaccines. Provenge represents autologous CD54+ cells which include monocytes and DCs. These are enriched from peripheral blood by leukapheresis and loaded *ex vivo* with a recombinant fusion protein construct consisting of full-length Prostatic Acid Phosphatase (PAP) linked to a full-length GM-CSF. This cytokine is included to target the fusion protein to APCs through the GM-CSF receptor (CD164), where it stimulates T cell immunity to PAP. Of all the antigen platforms currently available in late-stage development, PAP is the only defined target that may be considered organ-specific, or even tumor-specific, as it has no evidence of expression outside of the tumor and the tumor-bearing organ. Although this system targets a single antigen, the immune response engendered may be polyvalent if it recognizes more than a single PAP epitope [135]. 

Provenge showed OS benefit to patients in three double-blind randomized phase III clinical trials: D9901 [121,136]; D9902a [136] and IMPACT [135]. The D9901 trial enrolled 127 patients with asymptomatic metastatic hormone refractory prostate cancer (HRPC) randomized at a 2:1 ratio. The median survival time for patients treated with Provenge was 25.9 months compared to 21.4 months for the placebo-group patients. The difference in OS was statistically significant (*p* = 0.01). The D9902a trial was designed like the D9901 trial but enrolled 98 patients. The median survival time for patients treated with Provenge was 19.0 months compared to 15.3 months for placebo-treated patients, but did not reach statistical significance. The IMPACT trial served as the basis for licensing approval of Provenge by the FDA [2]. This trial enrolled 512 patients with asymptomatic or minimally symptomatic metastatic HRPC randomized at a 2:1 ratio. The median survival time for Provenge patients was 25.8 months compared to 21.7 months for the placebo-group patients. The difference in OS was statistically significant (*p* = 0.032). The prolongation of the median OS of vaccinated patients by 4.1-month is numerically the greatest survival benefit that has been seen in the treatment of HRPC. Sipuleucel showed no effect on time to progression in asymptomatic or minimally symptomatic metastatic castrate-resistant patients. 

The FDA approval of Provenge [2] has been a long process of clinical trials which began in 1997. Since the proposed procedure has had to be kept “locked” during the process of FDA approval, the antigen loading of DCs has been unchanged for 13 years. Within this period, scientiﬁc knowledge on DCs and their regulatory mechanisms have been greatly expanded. Therefore, the procedure for Provenge may be outdated and the efﬁcacy of Provenge may be unsatisfactory when based on current standards. 

Further encouraging proof of principle results came from a Phase I trial on glioblastoma, in which patients were treated with DCs loaded with autologous acid-eluted tumor peptides, leading to increased time to tumor progression and OS [137]. Despite this, crucial questions remain: why are the clinical responses still scarce and what can be done to improve the efficacy of vaccination?

#### 5.2.3. Future DC-Based Cancer Vaccine Therapy

It has been shown that DCs, when isolated from cancer patients, can have major dysfunctions. They can have a reduced capacity to stimulate an immune response than do DCs isolated from healthy donors [138,139]. DCs are modified by the tumor micro-environment through several mechanisms that protect the tumor from recognition by T cells. These mechanisms can in general be divided into four categories: (i) elimination of DC function by impairment of antigen capture, processing and presentation or lack of production/differentiation/maturation; (ii) induction of apoptosis in DCs; (iii) polarization of DC subpopulations into immuno-suppressive or tolerogenic cells and (iv) prevention of DC attraction to the tumor site by down-regulation of DC-attracting chemokines. 

Multiple stroma- and tumor-derived factors have been recognized to have the potential to alter the DCs at tumor sites or at a systemic level. All of these mechanisms result in a lack of T cell stimulation. A major challenge for successful DC-based cancer therapy consists then in overcoming the immune suppression induced by the tumor micro-environment. To this end, it appears of importance to identify and optimize the appropriate combination of molecular signals that results in the induction of potent and clinically significant anti-tumor immunity. Determining parameters of optimal DC loading with TAAs and of their activation is expected to be of major significance for improvement of clinical efficacy.

**Figure 4:**
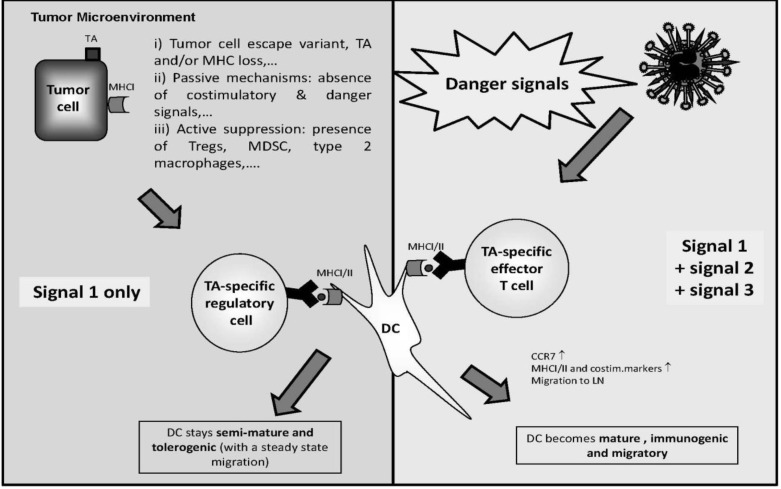
NDV drives dendritic cells (DCs) from tolerogenicity to immunogenicity (see the main text for more details).

The large variability in vaccine protocols, relating to antigen type, dose, injection frequency, and route of administration, among others, makes it difficult to compare clinical trials and finally to elaborate any precise conclusion from them. Despite the disappointing results of their first clinical evaluations, DC-based vaccines present a great potential for inducing anti-tumor immune responses to stabilize the disease. NDV offers new possibilities to optimize the *ex vivo* DC activation in order to maximize the quantity and quality of effector T cells and to minimize the development of Treg that undermines anti-tumor responses (Figure 4).

This should allow overcoming the obstacles due to the immuno-suppressive micro-environment induced by the tumor and allowing the recruitment and the functional activation of the vaccine-induced CTL.

## 6. Combining Systemic NDV Application with DC Vaccination

### 6.1. NDV-DC Therapy

To induce strong anti-cancer effects, an NDV-DC therapy is being developed by DeltaVir (Leipzig, Germany). It combines NDV and DCs in 2 sequential steps: (i) first, NDV is injected systemically; (ii) thereafter, vaccinations are performed via intradermal application of a DC vaccine loaded with NDV infected autologous tumor oncolysate. This second step is based on *ex vivo* generation of DCs which are derived from the cancer patient. These are instructed and activated with the help of the patients’ tumor lysate and with the use of NDV as oncolytic and stimulating agent.

The NDV-DC therapy combines immune conditioning via oncolytic NDV with DC-oncolysate immunization. It represents a new immunotherapeutic concept by its potential to increase innate immunity mechanisms and adaptive anti-tumor immune responses through priming of naive T cells and through re-stimulation of anti-tumor memory T cells. 

The studies with ATV-NDV revealed the importance of tumor cell viability and vaccine tumor cell number for clinical effectivity [89]. In the NDV-DC vaccine, tumor cell viability is replaced by DC cell viability. Such a vaccine is expected to be superior to ATV-NDV because it allows to initiate *de novo* tumor specific T cell responses from the pool of naive T cells.

### 6.2. Rationale for the Systemic Application of NDV

The rationale for the systemic application of NDV *MTH-68* is based on the effects described above: (i) the selective replication of the *MTH-68* virus in tumor cells, (ii) the oncolytic properties of NDVand (iii) the expected post-oncolytic immune effects to reinforce pre-existing anti-tumor immune memory of the cancer patient. It is also based on indirect effects: (i) the NDV-induced production of high levels of type 1 IFNs, (see Section 6.2.1); (ii) the Th1 polarizing and thus immune system conditioning effect of the virus-induced IFN-α and ß (see Section 6.2.2) and (iii) the helping effect of virus-induced T helper cells for the response to NDV-DC (see Section 6.2.3).

#### 6.2.1. Production of High Levels of Type 1 IFN

NDV is one of the viruses which induce a strong type 1 IFN response. This is important for cancer therapy since type 1 IFNs have direct anti-tumor activities through cell-cycle regulatory, anti-proliferative and anti-angiogenic effects (for a review, see [140]). Upon replication in infected cells, dsRNA and other viral RNA intermediates, whose exact structure is the subject of intensive research [141], are produced in the cytoplasm. These, via interaction with RIG-I, induce an IFN response in the infected cells. In addition to this autocrine type 1 IFN production, a paracrine mechanism of type 1 IFN and TRAIL induction by the NDV-HN protein has been described [45,46]. 

Plasmacytoid DCs (pDCs) have been identified as a minor cell population, which is responsible for the production of large quantities of type 1 IFNs upon NDV triggering through direct contact with these cells. The existence of these different pathways using autocrine and paracrine mechanisms [45,141] might, at least partially, explain the capacity of NDV to induce high levels of type 1 IFN since they are not only produced upon viral replication but also via contact of the virus with human peripheral blood mononuclear cells including monocytes. IFN-α plays a crucial role in the immune system. It up-regulates the expression of MHC class-I on the cell surface [41], skews differentiation of monocytes into DCs [142], stimulate the development and activity of DCs [143], enhances the proliferation of Th1 lymphocytes and is also important of the generation and survival of CTLs [51]. 

#### 6.2.2. Effect of Virus-Induced T Helper Cells on the Response to NDV-DC

Type 1 IFNs are considered to be major players for linking innate to adaptive immunity. They were first discovered for their innate anti-viral activity [144,145]. They activate DCs [146]—by promoting the expression of co-stimulatory molecules [142,147,148]—but also human blood monocytes by stimulating their differentiation into DCs [143]. Type 1 IFN-treated DCs can then prime T cells *in vitro* more effectively [144]. Such DCs also up-regulate expression of LN-homing CCR7 and exhibit stronger migratory capacity compared with DCs differentiated with other cytokines [149]. In addition, type 1 IFNs further promote cross-priming of CD8+ T cells via direct stimulation of DCs [150]. 

Type 1 IFNs also have an effect on the regulation of effector and memory cells by inhibiting their death [151]. Type 1 IFNs alone failed to promote Th1 commitment in human and mouse CD4+ T cells *in vitro*, although they induce STAT4 activation. The co-presence of IL-12 appears important for a strong CD4+ T cell response able to drive Th1 development [152,153]. The effect of type 1 IFNs on the generation of antigen-specific CD4+ T cells *in vivo* is expected to help the induction of an anti-tumor response during the vaccination steps of the NDV-DC therapy. 

#### 6.2.3. Th1 Polarizing and Immune System Conditioning Effect

There are other considerations, which corroborate the new concept, as for instance, the inhibitory effect of type 1 IFN on Th2 and Th17 differentiation of T cells. Cancer patients have often an immune system which is polarized towards a Th2 cytokine profile. Some subsets of leukocytes certainly exhibit antitumor activity, including CTLs and NK cells [154], other leukocytes, most notably mast cells, B cells, dendritic cells, granulocytes, and macrophages, exhibit more bipolar roles, by virtue of their capacity to either hinder or potentiate tumor progression [155,156]. CD4+ Tregs, Type 2 CD4+ T cells, Type 2 natural killer T cells, MDSCs, M2 or tumor-associated macrophages, B cells, and possibly mast cells promote tumor progression. In contrast, CD8+ T lymphocytes, type 1 CD4+ T lymphocytes, NK, type 1 natural killer T cells, M1 macrophages, and immune killer dendritic cells promote tumor destruction. Tumor immunity appears thus as a balance between immune mediators that promote tumor progression versus mediators that promote tumor rejection. 

Type 1 IFNs play a role in this balance since they inhibit secretion of Th2 cytokines (IL-4 and IL-5) and stimulate IFN-γ production [157,158]. Cancer patients may then profit from the systemic application of NDV since this has a conditioning effect towards a Th1 response. In addition, IFNs were also shown to enhance adaptive responses via direct effects on effector and memory T cells, particularly CD8+ T cells [159], by stimulating their proliferation. Systemic application of NDV exerts a conditioning effect on the patients’ immune system via inducing the production of type 1 IFN, it supports Th1 polarization of the antitumor immune response and favors the activation and expansion of T cells (including those which are tumor specific).

We conclude that NDV as OVs (i) upon systemic administration can modify the immune context and (ii) consequently can facilitate the generation of anti-tumor immunity. The systemic administration of NDV before DC vaccination is a new strategy for the treatment of cancer patients.

### 6.3. Rationale for the Use of DCs and NDV Oncolysate of Autologous Tumor Cells

The objective of this vaccination step is, via injecting activated and TA-presenting DCs, to generate *de novo* anti-tumor immune responses (from naive T cells) and to reactivate anti-tumor reactive memory T cells from the repertoire of pre-existing memory T cells, including partially anergized cells. Strong stimulatory signals are required since vaccine efficacy may be blunted by the immuno-suppressive milieu, which is characteristic for cancer patients. This is linked to numerous mechanisms such as the increased presence of Tregs, and inhibitory pathways such as the PD-1/PDL-1. The rationale for the vaccination with NDV-DC is based on the use of autologous DC, autologous tumor cells and NDV having oncolytic and immunostimulatory properties.

#### 6.3.1. Autologous DCs

The use of *ex vivo* generated DCs is expected to side-step the cancer-associated dysfunction of endogenous DCs and to deliver the key instructive signals needed for effective anti-tumor responses. Such information through DCs as APCs is important for the immune system to identify, via TAAs, the target (*i.e.*, autologous tumor, minimal residual disease, metastases) and for the T cells to associate such target antigens with danger signals derived from the virus. 

#### 6.3.2. Autologous Tumor Antigens

Candidate tumor antigens for vaccination include shared self-antigens including cancer/testis antigens and tissue differentiation antigens and unique (mutated) antigens [160,161]. The choice between these two types of antigens for vaccination could be viewed as a choice between inducing immunity (mutated antigens) or breaking tolerance and inducing autoimmunity (self-antigens). Shared antigens are attractive as they might allow us to establish “generic” vaccines. 

However, the enthusiasm for these antigens might be dampened by the 2 following reasons: (i) the repertoire of T cells might be deleted of high avidity clones through negative selection by shared antigens in the thymus [162] and (ii) the existing memory T cells might be polarized in a Tc2 orientation via the existing repertoire of Th2 cells [163,164]. 

Unique (or mutated) antigens are postulated to be superior to shared antigens [160,161]: their T cell repertoire is not deleted and they should be recognized as nonself by the immune system, as is the case with viral antigens [160,161].

The use of autologous tumor cells enables a close match between the TAAs of the vaccine and those of the patient’s tumor and includes common and individually unique TAAs [165]. Unique TAAs represent the only true, tumor-specific antigens that are not expressed by any normal tissue. An important additional feature of unique TAAs is their potential resistance to immunoselection in cases in which the mutated protein is crucial to the oncogenic process. In conclusion, unique TAAs are important for the induction of long-lasting tumor-specific protective immunity. 

#### 6.3.3. The Oncolytic and Immunostimulatory Properties of NDV

The dsRNA replicating forms, which are synthesized in the cytoplasm during NDV replication, are recognized by TLR3 and RIG-I in a cell type and pathogen type-specific manner [19,43]. It has been described that also the uncapped 5' end of the viral RNA genome is recognized by RIG-I. Studies in RIG-I deficient mice revealed that conventional DCs from these mice have impaired type 1 IFN responses after infection by NDV. In contrast, pDCs from these mice [19] were able to respond to NDV by production of high amounts of IFN-α [166]. Thus, the TLR system appears to be required for pDCs to induce the anti-viral response while, for conventional DCs, RLRs are critical to sense NDV. 

It was shown that dsRNA, which is present in the apoptotic bodies of virus-infected dead cells, is recognized by CD8α+ DCs that have high expression of TLR3 [167]. This promotes cross-priming of T cells to virus infected cells [168]. The release of high amounts of IFN-α by PBMC is due to activation of DCs (both myeloid and plasmacytoid) but also of monocytes. In DCs, the infection by NDV will lead to an abortive cycle of viral replication. However, some viral genes will be transcribed. The viral genome itself induces a strong type 1 IFN response in these cells, which will stop the viral replication.

Maturation of DCs will be accomplished by NDV infection via activation of pathogen sensing pathways through PRRs, including the TLR- and RLR-pathways, which are mentioned above. Very interestingly, a recent study shows that NDV favors development of type 1 DCs, also called «DC1» [169]. These up-regulate CXCL10, recruit and activate NK cells, instruct Th1 cytokine production, reduce Treg frequency [170], induce stronger CTL activation and support superior antigen cross priming [171]. 

When DCs were transduced with IFN-α prior to *in vivo* inoculation, their ability to migrate and survive was enhanced compared with control transduced DCs in human clinical trials [172]. Together, these findings support the use of NDV as strong inducer of type 1 IFN during DC differentiation. 

It is now clear that IFN-α has an important adjuvant function in the immune response. This effect could explain why most live viruses elicit strong immune responses whereas viral peptides are poorly immunogenic or tolerogenic unless supplemented with exogenous adjuvant. 

NDV, via induction of IFN-α, has been shown to induce in NK cells and monocytes TNF-related apoptosis-inducing ligand (TRAIL) and cell-mediated cytotoxicity [48]. Interestingly, there is a cross-talk between the IFN signalling pathway and TRAIL [173]. This can explain the fact that, in monocytes, NDV, via IFN-α, up-regulates the expression of TRAIL, which mediates their tumoricidal activity [48].

Type 1 IFN has been also reported to induce the IL-12 receptor-α chain in T cells [174]. Together with IL-12, IFN-α polarizes the T cell towards a cell mediated Th1 response characterized by DTH and CTL activity. In addition, IFN-α induces the up-regulation of molecules, which are important for antigen-recognition (e.g., human leucocyte antigen (HLA)) and cell-cell interaction (e.g., cell adhesion molecules [41]).

The role of type 1 IFN in the induction of the immune response is reinforced by its function as link between innate and adaptive immunity [175]. The innate immune response does not only provide the first line of defence against danger but also instructs the adaptive immune system to mount a fitting response [176].

The first published danger model of immunity [104] proposed only one mechanism for immune recognition of danger: that perceived by DCs upon release of cellular contents following necrosis of a diseased cell in its neighbourhood. This model predicts a superior effect of a lytic as opposed to a non-lytic virus in the treatment of tumors, because tumor cells necrotically destroyed by the virus would be phagocytosed and perceived as dangerous by DCs. In such a process, these professional APCs would process TAs, become activated and present processed TA peptides to T cells for cognate interaction and induction of an immune response. Thus, the rationale of this NDV-DC vaccine is to use autologous DCs and to instruct them with autologous tumor cell lysate which expresses multiple TAs (as signal 1 or antigen signal) and multiple danger signals (for the generation of the signals 2 (co-stimulation) and 3 (polarisation). Finally, the use of an OV allows us to lyse the tumor cells, thereby accelerating uptake of the tumor antigens by the DCs.

In conclusion, the NDV-DC vaccine, which combines autologous DCs with oncolysate from NDV infected autologous tumor, shows excellent prerequisites for superior activity. In comparison to classical DC therapies, the combined NDV plus NDV-DC approach is also new. “Classical” DC therapies have mainly employed DCs pulsed with TAs and matured by exposure to cytokine cocktails. These DCs are sufficient for T cell activation, but they may not be adequate to provide continuous co-stimulation to mount and maintain a pro-inflammatory immune environment and to recruit additional effector components. 

Since an existing tumor creates an immuno-suppressive environment, successful DC-based cancer vaccines should be prepared to be able, via immunization, to prime a strong and persistent primary immune response against tumor cells and to re-stimulate tumor-specific memory T cells. 

Even if the *ex vivo* antigen-loaded DCs would not directly activate naive T cells as efficiently as DCs that naturally acquire antigen *in vivo*, the combination of NDV and tumor antigens with DCs make these cells as carriers of TAs (signal 1) and of danger signals (signals 2 and 3) resistant to tolerogenic effects of the microenvironment. That appears to be a way to target tumor antigens and danger signals also to endogenous DCs. 

The combination of NDV with DCs can allow for a continuous supply of processed tumor antigen and immune-stimulating molecules with redundant and pleiotropic activities. It is therefore expected to provide more robust and persistent anti-cancer immunity *in vivo*. Numerous investigations have demonstrated that DCs can be simultaneously modified with multiple genes and/or immune factors [177,178]. However, it is unlikely that modification of DCs by a single factor, or engagement of a single effector population will be sufficient to ensure a successful cancer immunotherapy. 

Recently, NDV has been suggested in tumor therapy to help not only to stimulate T effector cells but also to block Treg cells, thereby alleviating a brake to anti-tumor activity [179]. Inactivated Sendai virus particles (paramyxoviruses like NDV) were reported to eradicate tumors by inducing immune responses through blocking Tregs [180].

Clinical trials of DC-based cancer vaccines have revealed that activated NK cells are better predictive of vaccine efficacy than CTL responses [181,182]. Accordingly, future clinical trials may benefit from the inclusion of NK cells in addition to CTL. NDV was demonstrated to induce tumoricidal activity in NK cells by binding to NKp46 receptors and initiating activation signals leading to cytotoxic activity and IFN-γ production [183]. The vaccine NDV-DC is expected to provide NK cell activation and to support activation of cancer-reactive T cells. The addition of viral oncolysate to DC appears as safe [184]. 

The strategy based on the use of oncolytic NDV, autologous tumor material and DCs appears to be sufficient for delivery of TAs, co-stimulatory molecules, and environmental signals. The use of oncolysate provides TAs directly to the cytosol of DCs after pinocytosis. This is expected to result in the induction of T cells with desirable effector functions and tumor-relevant homing properties. Combining DC vaccination with NDV oncolytic virotherapy might paves the way for a new strategy of cancer immunotherapy. This includes patients with tumors having poorly-defined rejection antigens, after primary or secondary tumor resection (with available tumor material). This group of patients is most likely to benefit from vaccination in an adjuvant situation.

## 7. Conclusion

**Figure 5:**
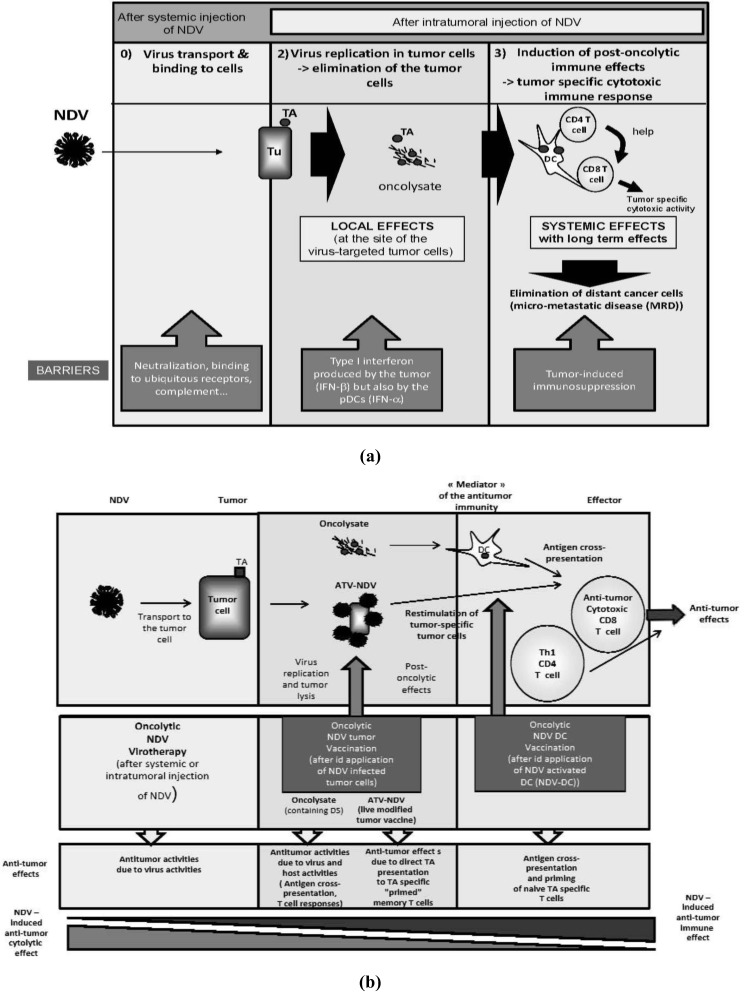
(**a**) The expected three steps after *in vivo* application of NDV; (**b**) Strategies to enhance NDV-induced anti-tumor immune activities (see the main text for more details).

During the last 6 decades, NDV has been tested as an agent against cancer. From the beginning, the experimental studies with NDV were performed in animals but also in humans. Results were published as case-reports and as prospective non-randomized clinical studies. These trials were based on the oncolytic properties of the virus, either directly *in vivo* (oncolysis *in vivo*) or *ex vivo* (by generating oncolysates). The observations made over this long period demonstrate the safety and potential of NDV as a new biologic agent against cancer (Figure 5a,b).

The selectivity of NDV for replication and cytolytic activity only in tumor cells has been attributed primarily to a defective type 1 IFN response which is a typical feature of tumor cells. However, in the recent years evidence has accumulated that also other features are of importance to explain the selective oncolytic activity of NDV [185,186,187,188]. For instance, a link was suggested to Rac1 expression and tumorigenesis [187]. NDV oncolytic activity against chemoresistant and apoptosis-resistant tumor cells was ascribed to pro-apoptotic activity [185,188]. The exact molecular mechanisms of tumor selectivity of NDV still need to be further analyzed and elucidated.

The development of the field of tumor immunology over the same time period changed the focus of the development of tumor treatment with virus, from virological considerations to more immunological ones, as shown by the pioneering work of William Cassel with vaccination with tumor lysates. The Heidelberg vaccine ATV-NDV and the associated basic and clinically applied research revealed new insights into the mechanism of antitumor immune properties of NDV and paved the way for further exploitation of the potential of NDV for anti-tumor vaccination. 

DC-based cancer vaccines have shown a parallel development, although being more recent. Their further development faces new challenges for the future but also new hopes for success. Data from recent clinical studies suggest that DC-based vaccines have the potential to induce strong anti-tumor responses and to prolong the survival of cancer patients.

New data reveal that NDV is a biologic agent to be combined with DC therapy to improve the efficacy of anti-tumor vaccination and to bring it to clinical efficacy [169]. In an attempt to increase DC-based potency and to improve immune responses following vaccination, the combination of systemic NDV treatment with vaccination with DCs loaded with NDV oncolysate is expected to recover or restore the dysfunction of DCs and T cells in cancer patients, to cause the migration of DCs into LNs, to stimulate effector rather than regulatory T cells and finally to confer to tumor-specific CTLs the ability to recognize and kill tumor cells. To this goal, two main approaches are combined: anti-tumor vaccination using DCs and use of OVs. The first road takes advantage of DCs as central cells of the immune system for the induction of an immune response, the second road takes advantage of the properties of certain viruses to lyse tumor cells and to induce long term post-oncolytic immune effects. The resulting NDV-DC therapy represents a new potential to be evaluated for the treatment of cancer patients.

The scientific data presented in this review provide new insights into the design and mechanism of function of NDV-DC. This vaccine works as a potent Th1 response mediator, favoring the induction of DC maturation, the release of pro-inflammatory cytokines and the improvement of antigen cross-presentation. All these steps are essential for the priming and activation of a CD8+ T cell-mediated tumor-protective immune response.

**Figure 6:**
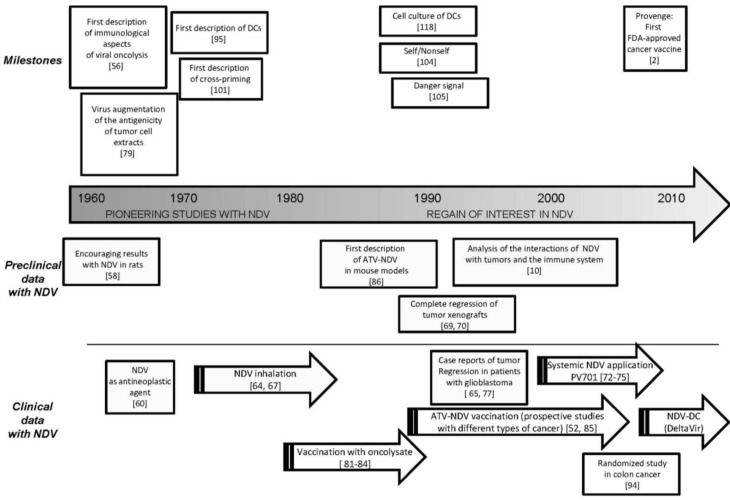
History of the development of cancer therapy based on NDV and the immune system (see the main text for more details).

Many milestones in nearly 50 years have shaped the scientific basis and clinical evaluation of the NDV-DC therapy (see Figure 6). It rests on the knowledge accumulated by many dedicated scientists and clinicians. Fifty years ago, much research was mostly intuitive without much information about cellular functions and interactions, or about the molecular nature of structures such as antigen-specific T cell receptor or of tumor antigens. Nevertheless, there already existed creative minds with ideas in the right direction.

The early clinical evaluations of the anti-tumor properties of NDV, which started in the 1960s and which were presented as case reports, were essentially experimental and observational. The vaccination studies, which were performed by William Cassel in melanoma patients with oncolysate, gave rise to interesting results, although their validity cannot be warranted [80,81,82,83,84]. The many prospective clinical studies and a randomized study with the vaccine ATV-NDV confirmed the potential of NDV as immunostimulating agent for inducing strong anti-tumor activity with clinical benefit for the cancer patients in terms of improvement of survival [94]. Only the future will reveal if the NDV-DC therapy will procure a survival advantage to cancer patients.
